# Enhancing the Feasibility of* Microcystis aeruginosa* as a Feedstock for Bioethanol Production under the Influence of Various Factors

**DOI:** 10.1155/2016/4540826

**Published:** 2016-07-31

**Authors:** Muhammad Imran Khan, Moon Geon Lee, Hyo Jin Seo, Jin Hyuk Shin, Tai Sun Shin, Yang Ho Yoon, Min Yong Kim, Jong Il Choi, Jong Deog Kim

**Affiliations:** ^1^Department of Biotechnology, Chonnam National University, San 96-1, Dun-Duk Dong, Yeosu, Chonnam 550-749, Republic of Korea; ^2^Department of Food Science and Nutrition, Chonnam National University, 77 Yongbong-ro, Buk-gu, Gwangju 550-757, Republic of Korea; ^3^Research Center on Anti-Obesity and Health Care, Chonnam National University, San 96-1, Dun-Duk Dong, Yosu, Chonnam 550-749, Republic of Korea; ^4^Department of Environmental Oceanography, Chonnam National University, San 96-1, Dun-Duk Dong, Yeosu, Chonnam 550-749, Republic of Korea; ^5^Department of Refrigeration Engineering, Chonnam National University, San 96-1, Dun-Duk Dong, Yeosu, Chonnam 550-749, Republic of Korea; ^6^Department of Biotechnology and Bioengineering, Chonnam National University, 77 Yongbong-ro, Buk-gu, Gwangju 550-757, Republic of Korea

## Abstract

*Microcystis aeruginosa*, a freshwater microalga, is capable of producing and accumulating different types of sugars in its biomass which make it a good feedstock for bioethanol production. Present study aims to investigate the effect of different factors increasing growth rate and carbohydrates productivity of* M. aeruginosa*. MF media (modified BG11 media) and additional ingredients such as aminolevulinic acid (2 mM), lysine (2.28 mM), alanine (1 mM), and Naphthalene acetic acid (1 mM) as cytokine promoted* M. aeruginosa* growth and sugar contents.* Salmonella* showed growth-assisting effect on* M. aeruginosa*. Enhanced growth rate and carbohydrates contents were observed in* M. aeruginosa* culture grown at 25°C under red LED light of 90 *μ*molm^−2^s^−1^ intensity. More greenish and carbohydrates rich* M. aeruginosa* biomass was prepared (final OD_660 nm_ = 2.21 and sugar contents 10.39 mM/mL) as compared to control (maximum OD_660 nm_ = 1.4 and sugar contents 3 mM/mL). The final algae biomass was converted to algae juice through a specific pretreatment method. The resulted algae Juice was used as a substrate in fermentation process. Highest yield of bioethanol (50 mM/mL) was detected when* Brettanomyces custersainus*,* Saccharomyces cerevisiae*, and* Pichia stipitis* were used in combinations for fermentation process as compared to their individual fermentation. The results indicated the influence of different factors on the growth rate and carbohydrates productivity of* M. aeruginosa* and its feasibility as a feedstock for fermentative ethanol production.

## 1. Introduction

The depletion of fossil fuels due to continuous consumption throughout the world and their contribution in environmental pollution and global warming shifted the interests of researchers to explore sustainable, economical, and ecofriendly energy sources alternative to petroleum based fuels [1, 2]. Bioethanol is an excellent substitute to gasoline fuels as comparatively cheap, convenient, and environmentally safe transportation fuels [3, 4]. Nowadays scientists are taking keen interests in bioethanol production from renewable feedstocks [5]. Algae are photosynthetic organism producing considerable amounts of carbohydrates which can be converted to bioethanol through fermentation process [6]. Algae as a feedstock for bioethanol production have many environmental and economical advantages [7]. Microalgae possess unique features such as rapid growth [8], high CO_2_-absorbing capacity, no need of cultivable land, and nonedibility which make it preferable over other feedstocks. In addition, the absence of lignin in algal cell wall facilitates the pretreatment of biomass and reduces the cost associated with pretreatment [9]. Furthermore, the simple structure and the ability to endeavor harsh environment also give them preference [10]. Some algal species cause blooming in fresh waters, which have harmful effects on aquatic life [11].* Microcystis aeruginosa *is blooming in freshwater of rivers and lakes producing harmful toxin, microcystin which kills the aquatic life in that zone, and contamination of a catchment area. Hence, using* M. aeruginosa* as a feedstock will be not only novel, feasible, and economical for bioenergy generation but also helpful in removing the toxic blooms.

Despite the adventitious aspects of algae as a potential feedstock for bioethanol production, there had not been done an appreciable work in this field. The research in this area is limited [12, 13]. One of the major limitations in this subject is the comparatively lower sugar contents in algal biomass as compared to lipids contents; that is why algal biomass is mostly used for biodiesel production. The algal carbohydrates are also of great importance for bioethanol production and some algae species possess more carbohydrates contents than lipids.

In order to improve this technology, there is a need of huge amounts of carbohydrates rich biomass. The amounts of sugars in the algal biomass are the basic and major requirements for bioethanol production. Higher yield of bioethanol needs algal biomass with higher amounts of carbohydrates. Hence, growing carbohydrates rich algae is a good solution.

The quantity of carbohydrates produced by algae is completely dependent on photosynthesis. For efficient photosynthesis algae required moderate quantity of CO_2_, optimum light intensity, optimum temperature, and suitable nutrients composition [14, 15]. Photosynthetic pigments in the algae cells, such as phycocyanin and chlorophyll, also play major and key role in photosynthesis. Most greenish algae can produce high amount of carbohydrates because of their high chlorophyll contents. Our aim of this work was to investigate the optimum conditions and nutrient composition for growing* M. aeruginosa* with higher growth rate and carbohydrates contents in order to obtain a carbohydrates rich algal biomass for generating higher yield of bioethanol.

## 2. Materials and Methods

### 2.1. Media Preparation

*M*.* aeruginosa* was cultured in modified BG11 medium (MF medium) with increased amount of Dipotassium hydrogen phosphate (K_2_HPO_4_) and freshly added urea. Ingredients of MF medium (BG11 modified medium) were dissolved in natural lake water. pH of the medium was set to 7.02. Trace elements were added from stock solutions at 1 mL/L of the MF medium. Naphthalene acetic acid (NPA; 1 mM) was the cytokinin added to the medium. 2 mL of vitamin complex (BeecomhexaYuhan Corporation) was added per liter of the media. Lysine, alanine, NH_4_OH, glucose, and aminolevulinic acid (LA) were added as 2.28 mM, 1 mM, 1 mM, and 2 mM, respectively. The volume of alanine, lysine, and LA added was 5 mL/L of the media. Similarly, 5 mL NH_4_OH and 10 mL 1 mM glucose were added per liter of the media to the respective flasks.

### 2.2. Algae Culturing

Different strategies were applied to grow more greenish and dense algae in a short time. First algae were cultured at flasks level and then in small bioreactor. All the requirements of light, CO_2_, air, and continuous shaking were ensured. LED light was provided under 16/8-hour light/dark regime. First algae were grown both under blue and red LED light of same strength (70 *μ*molm^−2^s^−1^) to determine the most supportive wavelength for algae growth and after determination various intensities of the most supportive wavelength were tested for fast growth of* M. aeruginosa*. The flasks were kept in a shaking incubator at 100 rpm. Temperature was kept 25°C throughout the culture. Associative bacteria* Salmonella* (OD_660_ = 0.86) were added to the culture in its log phase at 50 mL/L for the purpose of assisting* M*.* aeruginosa *growth.

### 2.3. Biomass and Sugar Contents Analysis

Samples after every 24-hour intervals were taken and were analyzed for biomass and sugar contents. Two milliliters of sample were used to evaluate the algae growth by determining OD at 660 nm. The biomass was hydrolyzed with 5 M H_2_SO_4_ for one hour and the resulting hydrolysate was neutralized with 10 M NaOH and centrifuged. The carbohydrates contents in the centrifuged algal juice were determined by following the method of Miller, 1959 [16], for reducing sugar analysis with small modification. 1 mL of the sample was mixed with 1 mL of dinitrosalicylic acid (DNS) solution and was heated at 100°C on a heating block for 10 minutes. Thereafter, the samples were immediately cooled by placing in cold water and the absorbance was checked at 575 nm.

### 2.4. UV Treatment

It has been studied extensively that the carbohydrate contents of algae can be increased under stress conditions, so that harsh environment was created for* M. aeruginosa* by exposing it to UV for 5 hours before pretreatment in order to increase its carbohydrates contents. The difference in growth pattern was monitored and the morphology of UV-treated algae was investigated with an image analyzing system (Nikon, Japan).

### 2.5. Biomass Preparation and Fermentation

*M. aeruginosa *culture was dewatered very efficiently with electric flocculation. Electric field was applied to* M. aeruginosa* culture which concentrated the algae from water. As microalgae cells possess negative charge, algae can be separated from water by applying an electric field [17, 18]. The resulting concentrated biomass was treated with 0.05% TiO_2_ and 0.01% CaO and then subjected to microwaves for 2 hours. Cellic CTech2 (Novozymes) was used for breaking the algal cell wall components and to liberate the internal sugars contents to the external media. Cellic CTech2 was added at 0.02 mL/L of the algae at pH 4.5. After the addition of enzyme Cellic CTech2, biomass was kept in a shaking incubator at 50°C for 4 hours. Further saccharification of the biomass was made by acid hydrolysis at high temperature. The biomass was treated with 5 M H_2_SO_4_ followed by autoclaving at 100°C for one hour. The purpose of all of the pretreatment methods was to produce algal juice enriched with monomeric sugar contents (fermentable sugars). The final filtered algae juice after autoclaving was then used as a substrate for fermentation process.

Three different microorganisms were used for converting the sugars of the algae juice to bioethanol, that is,* Saccharomyces cerevisiae*,* Brettanomyces custersainus, *and* Pichia stipites*. These microorganisms were used both individually and in combination in order to investigate the efficient fermentation with maximum yield. Fermentation process was carried out in 3 L volume fermenter with 1 L algal substrate containing the respective media for the used microorganisms. Temperature was kept 27°C throughout the fermentation process. After every one-hour interval sample was taken and analyzed for alcohol contents by dichromate analysis method [19].

### 2.6. Statistical Analysis

SPSS was used for statistical analysis. All experiments were performed in duplicates and triplicates. Data are the mean values ± standard deviation. Data was analyzed using ANOVA. Statistical significance was set at *α* = 0.05.

## 3. Results and Discussions

Growth of* M. aeruginosa *was determined by measuring OD of the algae with a spectrophotometer at 660 nm. OD (optical density) is proportional to the density of the algal population in the suspension. An increased growth of* M. aeruginosa* was achieved by optimizing different parameters, such as temperature, light intensity, type of culture media, and additional nutrients. Light and CO_2_ supply was ensured throughout the culture. The effect of a parameter on algal growth was determined by the increase in biomass and sugar content of* M. aeruginosa *culture.

### 3.1. Growth Condition with Media

**Figure 1:**
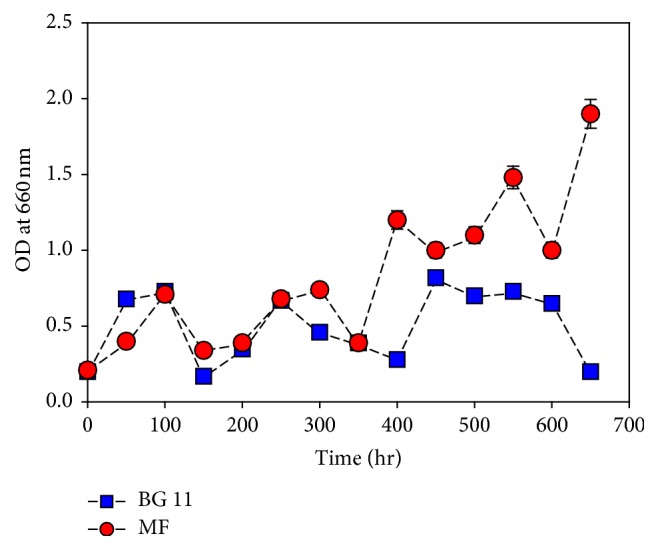
Effect of medium composition on* M*.* aeruginosa *growth. Data represent mean values of different experiments performed in duplicate ± standard deviation.

*M. aeruginosa *was cultured in both modified and original BG11 media. The modified BG11 medium showed effectiveness in supporting algal growth. The growth rate of* M. aeruginosa* was increased in the modified medium compared to that in the original BG11 medium (Figure 1). The modified medium was distinct from the BG11 medium in that the modified medium contained urea, an increased level of phosphate, Fe ion, and decreased Ca^2+^ (Table 1).* M. aeruginosa *cultured in the modified BG11 medium showed an increased OD and carbohydrate concentration in comparison to that cultured in the BG11 medium.

### 3.2. Growth Condition with Red/Blue Wavelength

**Figure 2:**
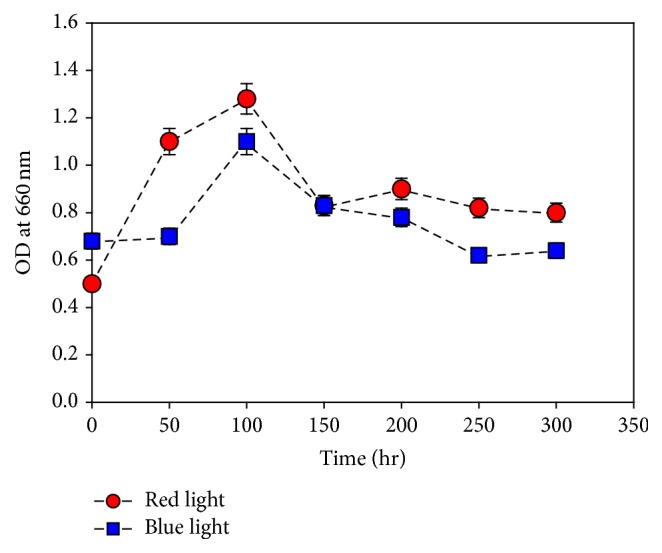
Effect of light wavelength on* M*.* aeruginosa *growth. Values are the averages of different experiments performed in duplicate ± standard deviation.

The light requirements of* M*.* aeruginosa* for photosynthesis and its effect on the growth of the algae were examined by culturing it under red and blue light. The growth of* M*.* aeruginosa* increased under red wavelength compared to that under blue wavelength (Figure 2). It has been studied experimentally that red light favors formation of cell walls and intercellular matrix, cell division, and accumulation of carbohydrates and increases the photosynthetic efficiency of unicellular green algae [20].

### 3.3. Growth Condition with Light Strength

**Figure 3:**
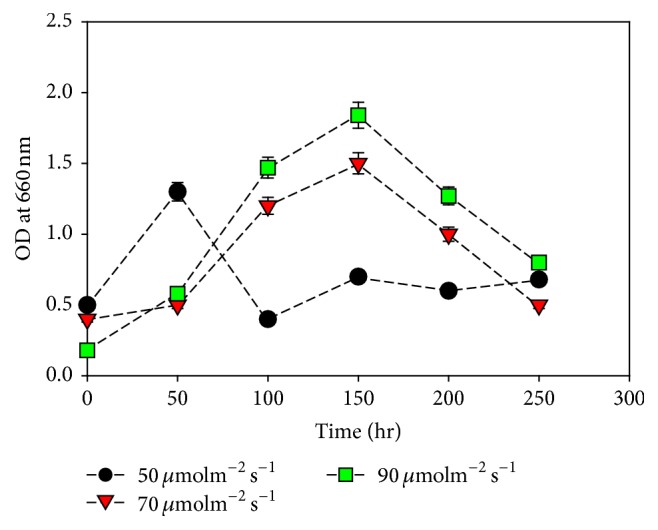
Effect of light intensity on* M*.* aeruginosa* growth. Values are the averages of different experiments performed in duplicate ± standard deviation.

*M. aeruginosa *culture was tested under different light intensities of red LED light that is 50 *μ*molm^−2^s^−1^, 70 *μ*molm^−2^s^−1^, and 90 *μ*molm^−2^s^−1^. The highest OD of* M. aeruginosa* was observed in the culture grown under 90 *μ*molm^−2^s^−1^ (Figure 3). Duration and intensity of light have a direct effect on photosynthesis and growth of microalgae. A constant light and dark period is necessary for growth of algae. Light period is required for photosynthesis and production of ATP and NADH while dark period is needed for synthesis of molecules essential for growth [21]. It has been experimentally proved that different weight of algae biomass is produced by culturing under different light intensities [22]. Although different algae species require different range of light intensities, most of the algae species show maximum growth in moderately high light intensity. Ifeanyi et al. 2011 [23] experimentally proved a rise in growth rate of* Aphanocapsa alga *cultured under light of 5000 lux which is about 90 *μ*molm^−2^s^−1^.

### 3.4. Growth Condition with Temperature

**Figure 4:**
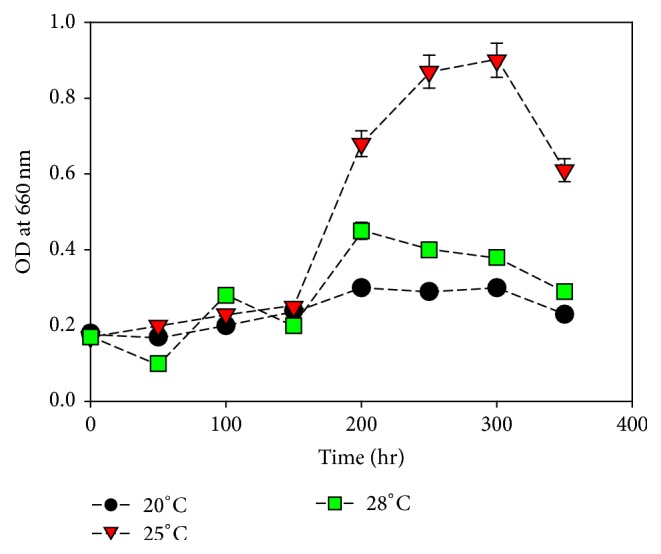
Effect of temperature on* M*.* aeruginosa* growth. Values are the averages of different experiments performed in duplicate ± standard deviation.

*M. aeruginosa *was cultured under different temperature that is 20°C, 25°C, and 30°C. The cultured grown very well at 25°C as compared to other cultures grown under various temperature ranges (Figure 4). Reduction in the growth rate was observed below and above 25°C. Algae species varies in the temperature requirements. Temperature has a key and crucial role in the growth of living organisms. Most of the algae species have their optimum temperature at which they grow to the maximum. Konopka and Brock (1978) [24] reported optimum growth of* Microcystis*,* Aphanizomenon,* and* Anabaena *at 25°C.

### 3.5. Growth Condition with Cytokines

**Figure 5:**
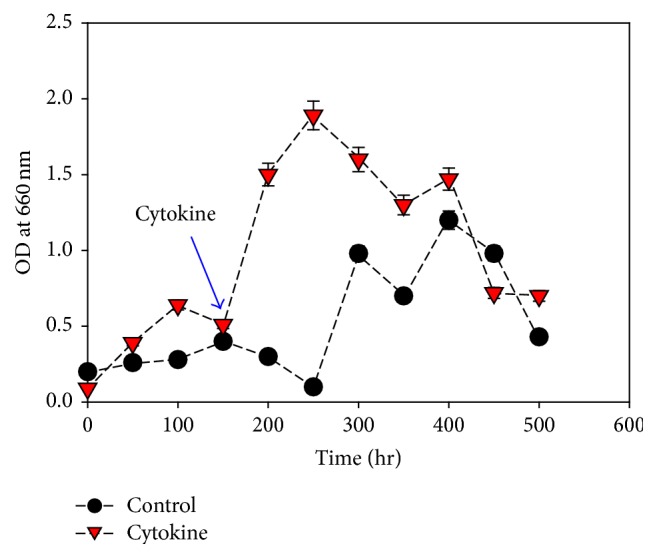
*M*.* aeruginosa* growth in presence and absence of cytokine. Values are the averages of different experiments performed in duplicate ± standard deviation.

Naphthalene acetic acid (NPA), synthetic auxin, was used as cytokines to stimulate* M. aeruginosa *growth. The growth rate of* M*.* aeruginosa *was increased upon the addition of NPA to the culture media. A difference was noted in the growth rate of the two cultures of* M. aeruginosa* with and without cytokines (Figure 5). Our results were assisted by Hunt et al. 2010 [25] who reported that NPA had a favorable influence on the growth of* Chlorella sorokiniana*.

### 3.6. UV Treatment

**Figure 6:**
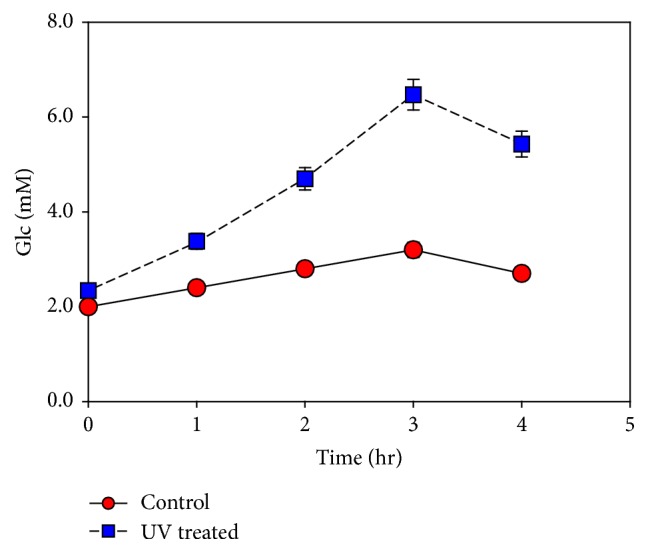
Effect of UV on* M*.* aeruginosa* sugar contents. Data represents mean values of different experiments performed in duplicate ± standard deviation.

**Figure 7:**
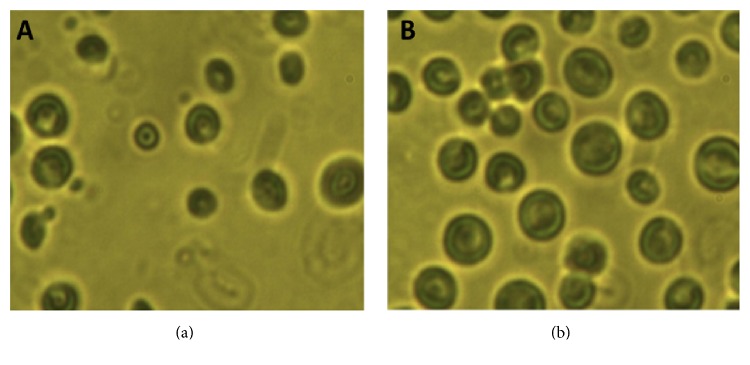
*M*.* aeruginosa *morphology under image analyzing system (Nikon, Japan) before and after UV treatment.

Carbohydrates contents were increased when* M. aeruginosa* culture was subjected to UV treatment for 5 hours (Figure 6). Morphological changes were observed in* M. aeruginosa*, after exposure to UV. Changes in the cell shape were examined with the help of an image analyzer system (Figure 7). With exposure to UV radiation for a specific duration of time, the algae cells modify their metabolic pathways for rapid synthesis of carbohydrates and lipids.

### 3.7. Optimal Growth Condition with Ingredients

OD and reduced sugar contents were measured in the samples drawn from each flask and bioreactor rafter every 24 hours.

#### 3.7.1. Flask Level Culture

**Figure 8:**
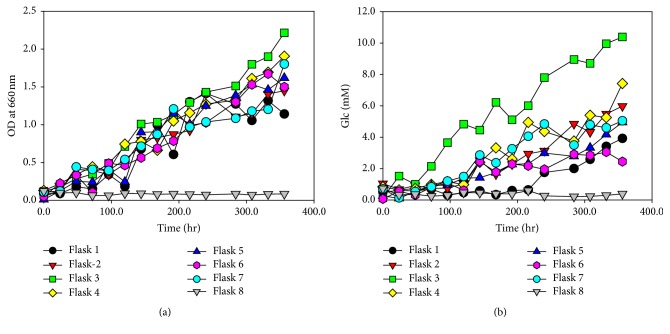
Flask level culture. (a) OD biomass (per mL) in different flasks versus time. (b) Glucose concentration (mM/mL) in different flasks versus time. Data represents mean values of different experiments performed in triplicate ± standard deviation.

Media ingredients in flasks are shown in Table 2. The highest yield of carbohydrates (10.0 mM) and biomass (OD_660 nm_ = 2.2) were obtained in flask 3 while the lowest were found in flask 8 (Figure 8). Flask 3 contained lysine,* Salmonella, *and LA added to MF medium which favored high growth rate of* M. aeruginosa*. NH_4_OH showed growth retarding effect but in combination with LA and* Salmonella* it becomes less effective in growth retardation in flask 7. Flask 4 showed higher OD and carbohydrates contents than all other flasks after flask 3 due to the presence of salmonella and lysine but it was lower in OD and sugar contents than flask 3 due to the absence of LA. Amino acids can be transformed to polyamines, such as spermidine, diaminopropane, and cadaverine, by the action of microorganisms which are crucial in enhancing algal growth [26] that is why amino acids alanine and lysine were added to* M. aeruginosa* culture. LA acts as a precursor for photosynthetic pigments in bacteria and blue green algae, probably through the tetra pyrrole biosynthetic pathway [27]. Some bacterial strains have beneficial effects on algal growth; studies have revealed the association and the beneficial exchange of nutrients between some algae and bacterial species [28]. Bacteria may improve algal growth in several ways. They may provide extracellular growth stimulating factors in the media, which may have a beneficial effect on the algal photosynthesis and growth. Bacteria also possess the ability to degrade various nitrogenous compounds, which can be readily used by algae [29]. Some bacteria produce phytohormones, the polyamines, which are very important for algal and plant growth [30]. Specific bacterial strains possess the ability of producing and releasing vitamins to the external medium which are very important for algal growth as they require exogenous vitamins for their growth [31].

#### 3.7.2. Algae Culture in Bioreactor

Algae were cultured in 20 L bioreactor under the above tested conditions of maximum growth rate and carbohydrates contents. Ingredients composition in bioreactors was kept like flask 3 as maximum growth rate and sugar contents were detected in flask 3. When OD of the culture reached 2.0 and sugar contents 10 mM then the culture was harvested as biomass for bioethanol production.

### 3.8. Fermentation

**Figure 9:**
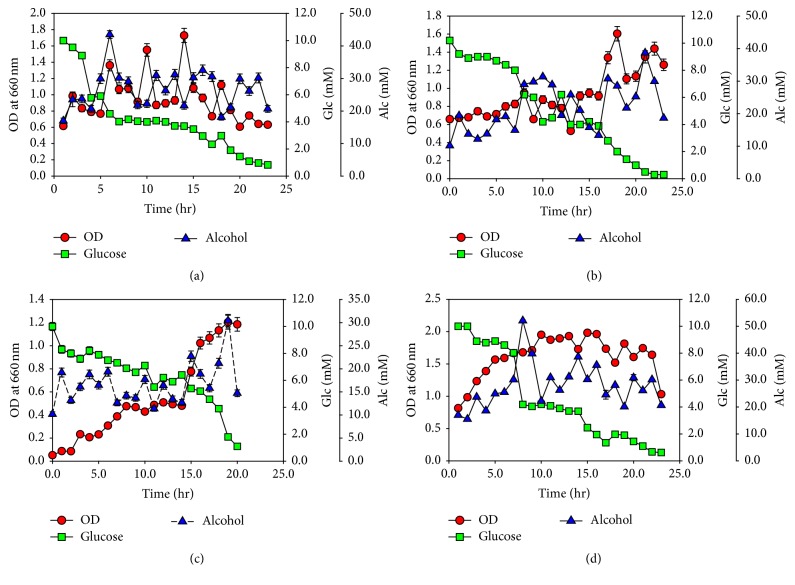
Fermentation of algae juice to bioethanol by three different microorganisms (alcohol contents/mL). (a) Fermentation by* Brettanomyces custersianus*. (b) Fermentation by* Saccharomyces cerevisiae*. (c) Fermentation by* Pichia stipitis*. (d) Combine fermentation. Data are the mean of values of different experiments performed in triplicate ± standard deviation.

As a result of the above used different conditions sugar rich algal juice was prepared and was used as a substrate for fermentation.* S. cerevisiae, B. custersainus,* and* P. stipites* were tested for their abilities of fermenting the algal juice to bioethanol. The highest yield of bioethanol (50 mM/mL) was noted when the three different microorganisms were used in combination. Individually* B. custersainus *showed higher yield of fermentative ethanol as compared to* S. cerevisiae *and* P. stipites* (Figure 9). The reason of* B. custersainus* higher bioethanol yield may be due to its ability of fermenting a wide range of sugars including cellobiose and maltose, [32] while* Saccharomyces cerevisiae*, the most commonly used ethanol producing microorganism, is unable to hydrolyzed pentoses. Similarly,* P. stipites* has the ability of converting xylose and rhamnose to ethanol but unable to act on other types of sugars [33] and the carbohydrates composition of* M. aeruginosa *includes different types of sugars [34] such as glucose, sucrose, cellobiose, maltose, ribose, lactose, rhamnose, galactose, and xylose (Table 3).

Algae are recently being explored as a renewable and sustainable feedstock for biofuels production [35]. However, at commercial level this technology needs more improvements and advancement for producing huge volumes of algal biomass and efficient pretreatment and conversion to bioethanol to compete the prices of fossil fuels [36]. Algae as a substrate for bioethanol production have not attracted admirable interest of researcher. Majority of algal species have relative low carbohydrates content except certain species which have relatively high sugar contents. However, this drawback can be overcome by increasing algal carbohydrates contents through media composition and applying stress condition to algae culture [37].* M. aeruginosa* contains a verity of sugars, which can be utilized for bioethanol production. Microalgae enriched with carbohydrates can produce 46,760–140,290 (L/ha) ethanol yield greater than any other feedstock [38]. In the present study, we focused on optimizing different parameters that enhance* M. aeruginosa* growth rate and sugar contents and efficient conversion to bioethanol.

## 4. Conclusion

*M. aeruginosa *possesses different types of sugars in its biomass. The higher growth rate of* M. aeruginosa* is crucial in increasing its carbohydrates contents. Lysine,* Salmonella*, cytokine, and LA have growth promoting effect on* M*.* aeruginosa* growth rate and carbohydrates productivity.* M*.* aeruginosa* was cultured in short time with enhanced growth rate and increased quantities of carbohydrates. A higher yield of bioethanol was obtained from the sugar rich algal juice by the combined fermentation process. Our results demonstrate the feasibility of* M. aeruginosa* as feed stock for bioethanol production.

## Figures and Tables

**(a):** 

Components	BG11 medium	Modified medium
NaNO_3_	1.5	1.5
K_2_HPO_4_	0.04	0.8
Ferric ammonium citrate	0.006	0.04
CaCl_2_·2H_2_O	0.036	0.001
MgSO_4_·7H_2_O	0.075	0.075
Na_2_CO_3_	0.02	0.02
EDTA	0.001	0.001
Citric acid	0.006	0.006
Urea	0	0.2
Trace metal solution	1 ml	1 ml

**(b):** 

H_3_BO_3_	2.86 g
MnCl_2_·4H2O	1.81 g
ZnSO_4_·7H_2_O	0.222 g
NaMoO_4_·2H_2_O	0.39 g
CuSO_4_·5H_2_O	0.079 g
Co(NO_3_)_2_·6H_2_O	0.0494 g
Distilled water	1.0 L

**Table 2:** Flask level culture; absence and presence of different ingredients and bacteria in flasks. Flask 1 was taken as a control.

Flasks	MF	Lysine (2.28 mM)	NH_4_OH (1 mM)	*Salmonella *(O.D = 0.86)	Algae seed (O.D = 0.12)	Glucose (1 mM)	LA (2 mM)	Alanine (1 mM)
1	200 ml	—	—	—	20 ml	—	—	—
2	200 ml	1 ml	—	—	20 ml	—	1 ml	—
3	200 ml	1 ml	—	10 ml	20 ml	—	1 ml	—
4	200 ml	1 ml	—	10 ml	20 ml	—	—	—
5	200 ml	—	1 ml	—	20 ml	1 ml	—	1 ml
6	200 ml	—	—	10 ml	20 ml	—	—	—
7	200 ml	—	1 ml	10 ml	20 ml	1 ml	1 ml	1 ml
8	200 ml	—	1 ml	—	20 ml	—	—	—

—, absence.

**Table 3:** Carbohydrates composition of *Microcystis aeruginosa*.

Sugar type	Quantity (*μ*g/500 mg)
Arabinose	20.43
Cellobiose	232.05
Galactose	262.64
Glucose	1912.66
Lactose	0.88
Maltose	57.64
Mannose	2.38
Rhamnose	893.53
Ribose	118.37
Sucrose	3496.85
Xylose	343.65
